# A case of membranous nephropathy complicated by autoimmune hepatitis and primary biliary cholangitis

**DOI:** 10.1097/MD.0000000000042770

**Published:** 2025-08-01

**Authors:** Xiwen Lei, Rui Dong, Jing Sun, Miaoxia He, Xiaobin Mei

**Affiliations:** a Department of Pathology, The PLA Naval Medical University, Shanghai Changhai Hospital, Shanghai, China; b Department of Nephrology, The PLA Naval Medical University, Shanghai Changhai Hospital, Shanghai, China.

**Keywords:** autoimmune hepatitis, membranous nephropathy, methylprednisolone, overlap syndrome, primary biliary cholangitis, rituximab

## Abstract

**Rationale:**

Membranous nephropathy (MN) is an important and common cause of nephrotic syndrome in adults. Overlap syndrome is an infrequent clinical subgroup characterized by the coexistence of autoimmune hepatitis, primary biliary cholangitis or primary sclerosing cholangitis. Coexistence of MN and autoimmune liver diseases is rare. Several reports have documented the concurrent presence of multiple autoimmune diseases, suggesting a potential mechanism linking these disorders.

**Patient’s concerns:**

A 53-year-old male patient diagnosed with phospholipase A2 receptor-related MN accompanied by recurrent increases in aminotransferases during treatment.

**Diagnoses:**

The patient was finally diagnosed as MN with primary biliary cholangitis-autoimmune hepatitis overlap syndrome based on histopathological findings.

**Interventions:**

A combination of rituximab, oral methylprednisolone, and ursodeoxycholic acid.

**Outcomes:**

The patient achieved complete immunological remission and partial remission of proteinuria.

**Lessons:**

We encountered a unique case with autoimmune disease of both liver and kidney. Treatment plans should be tailored to each patient’s specific condition since there is no standard protocol. The pathological mechanisms of overlapping autoimmune diseases require further understanding.

## 1. Introduction

Membranous nephropathy (MN) is an immune complex-mediated disorder of kidney glomerulus; based on the cause, it can be grouped into 2 major forms. Idiopathic membranous nephropathy stands for the majority (80%) for unknown etiology, whereas secondary MN (20%) is linked to systemic disorders involving hepatitis B or C, Lupus Nephritis, neoplasia, or drugs like NSAIDs.^[1]^ Antibodies to phospholipase A2 receptor (PLA2R) expressed in podocytes are likely responsible for 80% of idiopathic membranous nephropathy.^[2]^ MN is pathologically characterized by apparent thickening of the glomerular capillary walls with deposits of immune complex and electron-dense deposits (i.e., spikes) appearing in the subepithelium under electron microscopes.

Previous studies have reported the coexistence of autoimmune hepatitis (AIH) and primary biliary cholangitis (PBC)/primary sclerosing cholangitis, all of which feature the characteristics of autoimmune diseases while having a low prevalence. The clinical manifestations of PBC–AIH overlap syndrome are diverse but may involve 3 aspects: a biochemical overlap manifesting elevated aminotransferases or increased levels of cholestatic liver biochemistry; an immunologic overlap with positive auto-antibodies alongside elevated immunoglobulin G (IgG); and a histologic overlap showing moderate to severe interface hepatitis and destructive lymphocytic cholangitis.^[3,4]^ The aim of treatment is to achieve and maintain histological remission, thereby preventing secondary cirrhosis and ultimately avoiding end-stage liver failure.

Overlap syndrome can be found in multiple autoimmune diseases such as diffuse systemic sclerosis and ulcerative colitis.^[5,6]^ Among patients with PBC–AIH overlap syndrome, autoimmune thyroid diseases and Sjogren syndrome rank as the first and second most common co-morbid autoimmune disorders.^[7]^ To our knowledge, about 9 cases have been reported as MN with PBC/primary sclerosing cholangitis-AIH overlap syndrome in the world since 2008. Herein, we report a case of a 53-year-old male diagnosed with MN, complicated by AIH and PBC.

## 2. Case presentation

**Figure 1.**
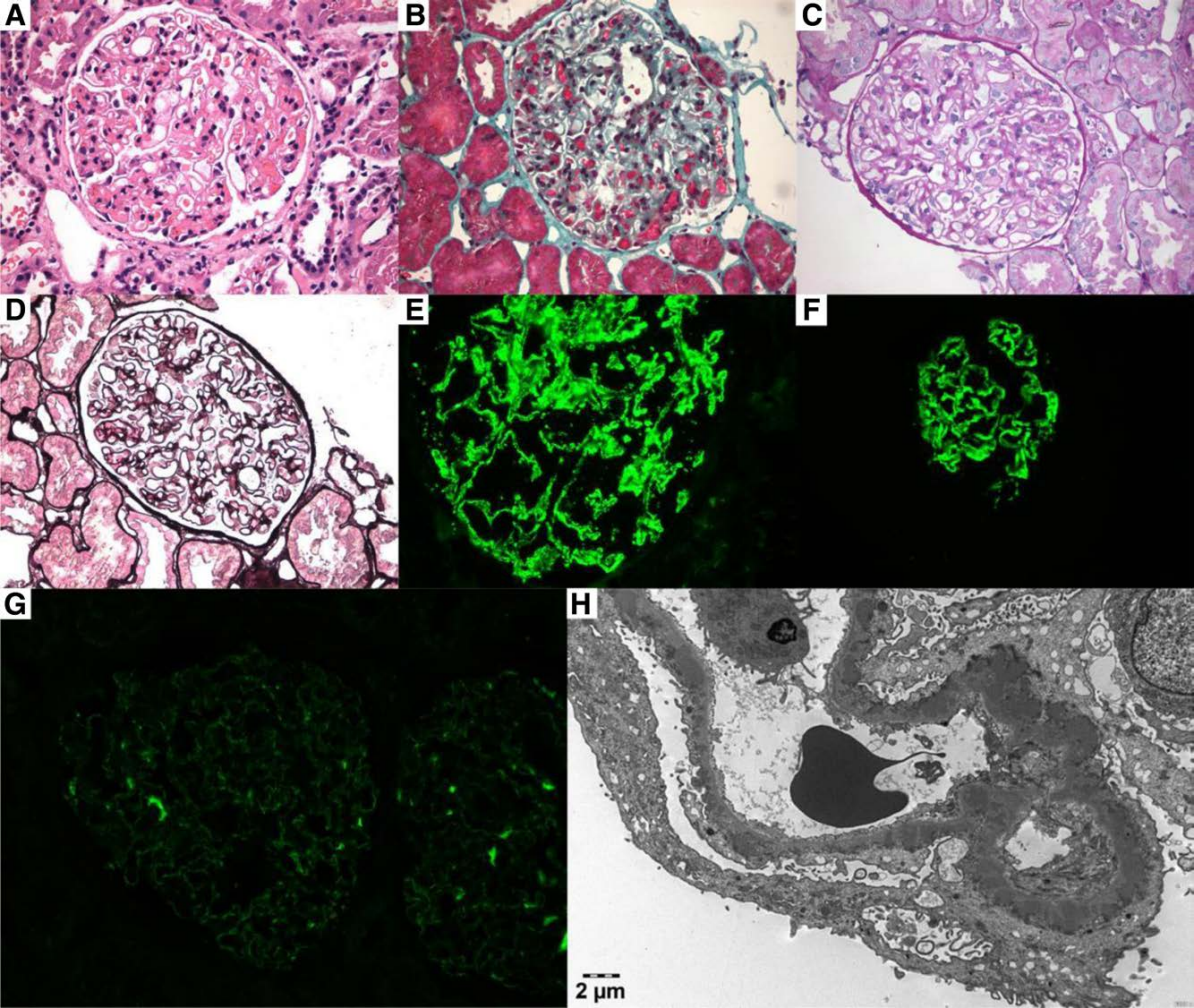
(A–D) Hematoxylin–eosin, Masson trichrome, periodic acid-Schiff, periodic acid-silver methenamine staining showed diffuse thickening of glomerular capillary walls (×400). (E–G) Immunofluorescence staining of kidney tissue (×400) for IgG, IgG4, and PLA2R was positive. (H) The electron microscopy image showed electron-dense deposits underneath the podocytes and extensive foot process effacement. PLA2R = phospholipase A2 receptor.

A 53-year-old male presented with edema in both lower extremities and foamy urine with no clear cause for 1 month. Sixteen days prior to his visit to our hospital, he had been diagnosed with proteinuria and hepatic insufficiency, given supportive treatment (details unknown), and his symptoms had been alleviated. Table 1 presents the patient’s clinical course. He sought further care at our hospital (day 1, June 20, 2023). The initial laboratory work-up was as follows: serum biochemistry revealed abnormal albumin (24 g/L), albumin/globulin (0.88), total cholesterol (8.3 mmol/L), low-density lipoprotein cholesterol (6.2 mmol/L), alanine aminotransferase (161 U/L), and gamma-glutamyl transferase (218 U/L); urinalysis showed elevated protein (13,245 mg/L), urine protein/creatinine ratio (9195.5 mg/g), and urinary albumin/creatinine ratio (7097 mg/g). Notably, the patient had a preexisting history of hypertension for over 6 months, which was effectively managed by taking allisartan and lercanidipine. A thorough physical examination showed a normal presentation, except for slight pitting edema. The patient had no known family history of heritable conditions associated with his clinical presentation. A renal biopsy (Fig. 1) was performed (day 21, July 10, 2023). MN stage II was diagnosed via pathological findings of the renal biopsy. Additionally, the circulating anti-PLA2R antibody (68.94 RU/mL) was positive; an anti-AMA-M2 test was first positive, then negative. Considering primary MN, the patient received 2 intravenous doses of rituximab (1 g each, 2 weeks apart). He was also administered 750 mg ursodeoxycholic acid (UDCA) once a day and 150 mg diammonium glycyrrhizinate 3 times a day.

**Table 1:** Diagnostic and therapeutic timeline.

Time point	Investigations	Diagnosis	Interventions	Therapeutic response
Preadmission	PRO, 4+. OB, 4+.	Proteinuria and hepatic insufficiency.	Supportive treatment (not documented).	Symptoms resolved.
June 20, 2023 (day 1)	ALB, 24 g/L. A/G, 0.88. TC, 8.3 mmol/L. LDL-C, 6.2 mmol/L. ALT, 161 U/L. γ-GT, 218 U/L. UP, 13,245 mg/L. UPCR, 9195.5 mg/g. UACR, 7097 mg/g.	Proteinuria, hepatic impairment and hypertension.	Allisartan (240 mg/d), lercanidipine (10 mg/d), ezetimibe (10 mg/d), α-keto acid analogues (0.14 g/kg/d).	No prior issues with hypertension control.
July 3–13, 2023 (days 14–24; first admission)	ALB, 23 g/L. A/G, 0.77. TC, 8.55 mmol/L. LDL-C, 5.62 mmol/L. ALT, 108 U/L. AST, 66 U/L. γ-GT, 194 U/L. IL-8, 22.76 pg/mL. anti-AMA-M2, positive then negative. anti-PLA2R antibody, 68.94 RU/mL. UP, 3393.0 mg/L. UPCR, 2506.1 mg/g. UACR, 1569 mg/g. UCa/Cr, 0.24 mg/g. Tf, 61.10 mg/L. κ-LC, 8.72 mg/L. λ-LC, 6.55 mg/L. UIgG, 37.9 mg/L. Urinary protein excretion/24 h, 8191.8 mg. Urinary albumin excretion/24 h, 6615.5 mg.	Nephrotic syndrome secondary to clinically suspected membranous nephropathy (PLA2R-positive), hepatic impairment and hypertension.	Renal biopsy, ezetimibe (10 mg/d), allisartan (240 mg/d), α-keto acid analogues (0.14 g/kg/d), ursodeoxycholic acid (750 mg/d), diammonium glycyrrhizinate (450 mg/d), first intravenous dose of rituximab (1 g).	Partial immunological remission of membranous nephropathy.
July 26–28, 2023 (days 37–39; second admission)	ALB, 20 g/L. A/G, 0.74. TC, 6.97 mmol/L. LDL-C, 4.75 mmol/L. γ-GT, 94 U/L. UP, 12,983.0 mg/L. UPCR, 8485.1 mg/g. UACR, 4597 mg/g. UCa/Cr, 0.24 mg/g. Urinary protein excretion/24h, 9357.0 mg. Pathological diagnosis: membranous nephropathy stage II.	PLA2R-associated membranous nephropathy and hypertension.	allisartan (240 mg/d), ezetimibe (10 mg/d), rivaroxaban (10 mg/d), α-keto acid analogues (0.14 g/kg/d), second intravenous dose of rituximab (1 g).	
January 19, 2024 (day 214)	ALB, 20 g/L. ALT, 228 U/L. AST, 112 U/L. ALP, 133 U/L. UA, 534 μmol/L. TC, 6.71 mmol/L. UACR, 1964.6 mg/g. Urinary protein excretion/24 h, 6898 mg. anti-PLA2R antibody, 7.94 RU/mL. Abdomen ultrasound: chronic liver damage.	PLA2R-associated membranous nephropathy, hepatic impairment and hypertension.	Polyene phosphatidylcholine and glutathione for hepatoprotection.	Limited hepatoprotective efficacy.
February 20, 2024 (day 246)	ANA (nuclear homogeneous type), 1:30. anti-dsDNA, 10 IU/mL. ANuA, 3.4 U/mL. ACA IgG, 6.8 PL-U/mL. anti-β2GP1, 15.5 RU/mL. ALB, 22 g/L. ALT, 282 U/L. AST, 230 U/L. ALP, 104 U/L. UA, 446 μmol/L. TC, 10.08 mmol/L.	PLA2R-associated membranous nephropathy, hepatic impairment and hypertension.	Liver biopsy, hepatoprotective and diuretic therapy.	Transaminase levels partially normalized.
March 20, 2024 (day 276)	Pathological diagnosis: Primary biliary cholangitis–autoimmune hepatitis overlap syndrome.	Autoimmune hepatitis, primary biliary cholangitis, PLA2R-associated membranous nephropathy and hypertension.	Oral methylprednisolone (32 mg once daily)	
April 16–18, 2024 (days 302–304; third admission)	ALB, 25 g/L. A/G, 1.09. ALT, 45 U/L. AST, 18 U/L. ALP, 65 U/L. γ-GT, 123 U/L. TC, 5.99 mmol/L. IL-8, 22.99 pg/mL. anti-PLA2R antibody, <2.0 RU/mL. UP, 1978 mg/L. UPCR, 2582.9 mg/g. UACR, 1949 mg/g. κ-LC, 11.6 mg/L. λ-LC, 7.72 mg/L. UIgG, 32.1 mg/L. Urinary protein excretion/24 h, 4450.5 mg.	PLA2R-associated membranous nephropathy, autoimmune hepatitis, primary biliary cholangitis and hypertension.	Allisartan (240 mg/d), ursodeoxycholic acid (750 mg/d), pantoprazole (40 mg/d), α-keto acid analogues (0.14 g/kg/d), rivaroxaban (10 mg/d), oral methylprednisolone (20 mg once daily), third intravenous dose of rituximab (1 g).	Complete immunological remission of membranous nephropathy and partial remission of proteinuria.

A/G = albumin/globulin, ACA = anticardiolipin antibody, ALB = albumin, ALP = alkaline phosphatase, ALT = alanine aminotransferase, ANA = anti-nuclear antibody, anti-AMA-M2 = anti-mitochondrial antibody, subtype M2, ANuA = antinucleosome antibody, AST = aspartate aminotransferase, dsDNA = double strand DNA, IgG = immunoglobulin G, IL-8 = interleukin-8, LDL-C = low-density lipoprotein cholesterol, OB = occult blood, PLA2R = phospholipase A2 receptor, PRO = protein, TC = total cholesterol, Tf = transferrin, UA = uric acid, UACR = urinary albumin/creatinine ratio, UCa/Cr = urine calcium-to-creatinine ratio, UIgG = urinary immunoglobulin G, UP = urinary protein, UPCR = urine protein/creatinine ratio, β2-GP1 = beta-2-glycoprotein 1, γ-GT = gamma-glutamyl transferase, κ-LC = kappa light chain, λ-LC = lambda light chain.

**Figure 2.**
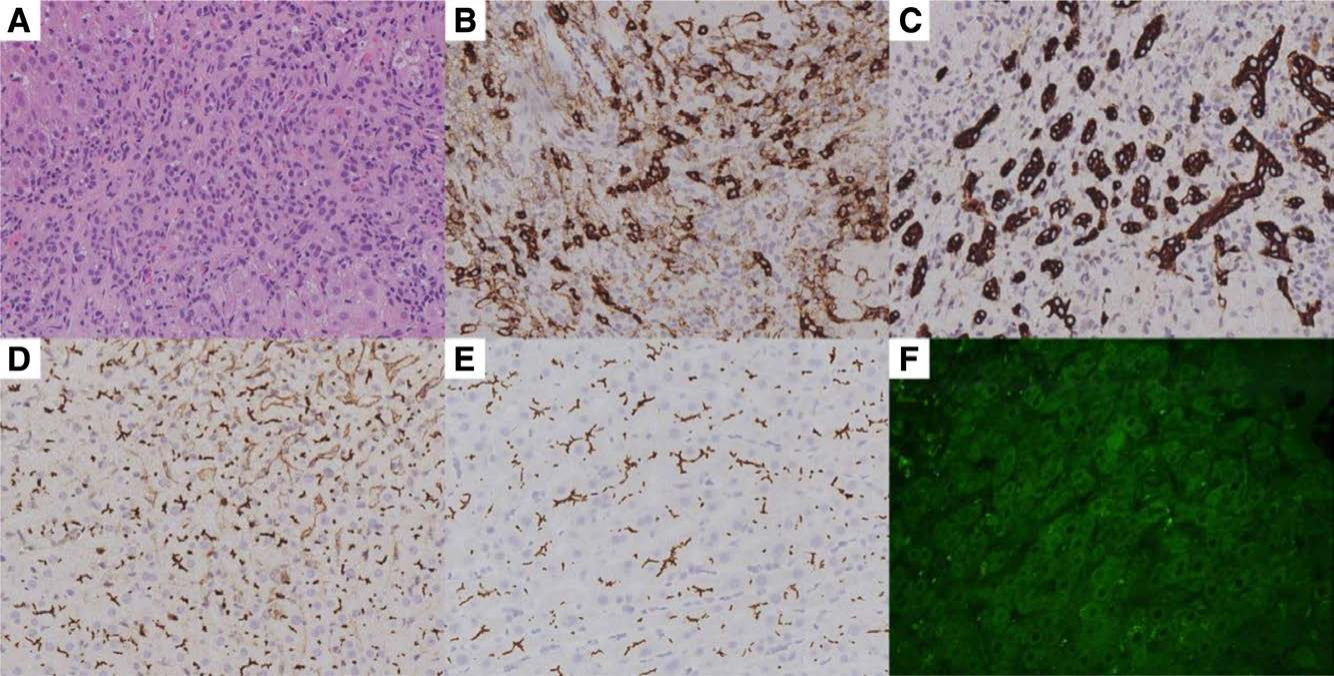
(A) Hematoxylin–eosin staining showed expansion of the portal areas with abundant lymphocytes and plasma cells, as well as proliferation of fibrous tissue (×200). (B) Immunohistochemical staining for CD38 showed significant infiltration of plasma cells (×200). (C) Immunohistochemistry staining for CK19 showed bile duct damage (×200). (D and E) Immunohistochemistry staining for CD10 and ABCB4 showed proliferation of bile capillary (×200). (F) Immunofluorescence staining of hepatic tissue for PLA2R was negative (×400). PLA2R = phospholipase A2 receptor.

Six months later, the patient was readmitted to the hospital owing to recurrent increases in aminotransferases. Reexamination showed abnormal blood and urine parameters: albumin 20.1 g/L, alanine aminotransferase 228 U/L, aspartate aminotransferase 112 U/L, alkaline phosphatase 133 U/L, uric acid 534 μmol/L, total cholesterol 6.71 mmol/L, triacylglycerol 3.19 mmol/L, urinary protein excretion/24 hours 6898 mg, urinary albumin/creatinine ratio 1964.6 mg/g. Serum anti-PLA2R antibody levels decreased to 7.94 RU/mL. An abdominal ultrasound indicated chronic liver damage. ANA (nuclear homogeneous type) 1:30, anti-dsDNA 10 IU/mL, AnuA 3.4 U/mL, ACA IgG 6.8PL-U/mL, anti-β2GP1 15.5 RU/mL, anti-AMA-M2, and anti-SLA/LP were negative. Administration of polyene phosphatidylcholine and glutathione did not yield any benefits for him. A liver biopsy (Fig. 2) performed on February 20, 2024 (day 246) indicated PBC–AIH overlap syndrome.

The patient was finally diagnosed as MN with PBC–AIH overlap syndrome. He was prescribed oral 32 mg methylprednisolone (later reduced to 20 mg) once a day with UDCA and received an infusion of 1-g rituximab 280 days after the initial dose. The delayed diagnosis of AIH and PBC was attributed to a neglect to spot early elevated values of aminotransferases and supposed positive auto-antibodies. Within 2 months, liver tests partially improved with alkaline phosphatase 65 U/L, alanine aminotransferase 45 U/L, aspartate aminotransferase 18 U/L, and gamma-glutamyl transferase 123 U/L. Despite the values of anti-PLA2R antibody being within the normal range (<2.0 RU/mL), the patient still had persistent proteinuria with a urine protein excretion of 3190 mg per 24 hours (day 423, August 15, 2024).

## 3. Discussion

Currently, MN, AIH, and PBC are all recognized as autoimmune disorders, distinguished by the presence of in situ immune complex deposits and/or circulating auto-antibodies that correlate with distinct histopathological changes. The pathogenesis of MN remains unclear; however, it is widely accepted that the primary driving force lies in immunological injury mediated by autoantibodies. Over the past few years, there has been a growing discovery of novel primary MN-related antigens, such as THSD7A, NELL1, SEMA3B, PCDH7, HTRA1, and NTNG1,^[8]^ whereas PLA2R is the most common target used in clinical practice. Both AIH and PBC include the production of autoantibodies as part of their disease development. One or more high-titer nonspecific antibodies are present in the serum of most AIH patients, including ANA, ASMA and anti-LKM. AMAs targeting pyruvate dehydrogenase complex-E2 autoepitope, considered as a critical factor in the pathogenetic process, are peculiar to PBC.^[4]^

In our case, the positive PLA2R antibody indicated that the development of MN may be associated with immunological injury mediated by autoantibodies. The diagnosis of AIH and PBC depends on the identification of autoantibodies and histopathological findings. The concurrent presence of these 3 disorders hints at a potential for a common genetic susceptibility and abnormal immune modulation. Genetic factors have been demonstrated as accounting for partial risk of the MN. Notably, several loci in HLA-DQA1, HLA-DR3, and PLA2R1 have emerged as robustly associated risk factors, with their influence varying based on the patient’s ethnicity.^[9]^ Similar findings have also been found in autoimmune liver diseases (AILDs). Susceptibility loci such as HLA-DR8, alongside non-HLA loci including IL12A, IL12RB2, IRF5/TNPO3, ORMDL3/IKZF3, MMEL1, and SPIB, were identified as contributing factors in PBC.^[10]^ Similarly, HLA-DRB1*0301 and HLA-DRB1*0401 were identified as susceptibility genotypes for AIH type-1.^[11]^ This finding highlights the pivotal role of these genetic triggers in predisposing diseases under an autoimmune context. In fact, these intricate genetic disorders manifest clinically in individuals, triggered by their responses to external risk factors. Autoimmune disorders arise due to loss of tolerance to self-antigens, significantly attributed to a complex interplay of environmental and hormonal factors, gut dysbiosis, infection, and even malignancy. Future studies that systematically incorporate HLA and broader genetic profiling in such cases may yield critical pathophysiological insights.

The standards of treatment for MN involve immunosuppressive therapy and supportive therapy aiming to protect the kidney and cardiovascular system. The former includes biologicals like rituximab and frequently-used immunosuppressants such as calcineurin inhibitors (cyclosporine and tacrolimus), cyclophosphamide and glucocorticoids.^[12,13]^ The main therapeutic choices for AIH are glucocorticoids and azathioprine.^[14,15]^ UDCA, a farnesoid X receptor activator, is dramatically used in all patients diagnosed with PBC as first-line therapy.^[4]^ Furthermore, the medication plan for MN with other overlapping AILDs needs further optimization. We assessed the patient’s risk as high risk following KDIGO 2021 guidelines and treated him with a combination of rituximab, oral methylprednisolone, and UDCA. This treatment achieved complete immunological remission of MN with anti-PLA2R <2.0 RU/mL and partial remission of proteinuria, as well as normalization of aminotransferases. B cell depletion therapy is considered when patients with AIH exhibit suboptimal response to first-line therapy. The safety and effectiveness of rituximab used in refractory AIH have been demonstrated in several reports.^[16–18]^ Despite clinical benefits to indicate that rituximab was effective for AIH, the treatment exhibits limited efficacy in PBC.^[19,20]^ Rituximab was selected over calcineurin inhibitors due to its more favorable profile, including intermittent dosing that improves adherence compared to daily regimens and reduced risk of hepatorenal toxicity-related discontinuation. Although PBC responses to rituximab remain inconsistent, a case report has documented the successful administration of rituximab in several autoimmune disorders including AIH-PBC overlap syndrome.^[21]^ This finding underscored the potential of rituximab as a viable therapeutic option for managing overlapping diseases with refractory AIH cases. Another case with the same diagnosis treated with UDCA, cyclosporin A, and methylprednisolone had similar persistent proteinuria until elevated doses of UDCA, prednisolone, and azathioprine were administered.^[22]^ There remains no standardized treatment consensus for these overlapping diseases, provoking individualized treatment to improve outcomes.

Another issue to be determined is the prognostic factors for overlap syndrome. The prognosis is currently dependent on the severity of the disease, the treatment options, and other factors. In our case, the patient achieved partial remission following active treatment, but long-time follow-up is still needed. Controversy persists over whether extrahepatic autoimmune diseases (EADs) affect AILDs outcomes. One study found that EADs do not affect the clinical outcome of AILDs,^[7]^ while another found that EADs lead to increased mortality among patients with AIH.^[23]^

In brief, intensified research is needed on the pathogenesis, treatments, and prognostic factors of coexisting autoimmune diseases. One intriguing challenge is whether a common target antigen or molecular link is shared between MN and AILDs. Meaningful progress requires deepened comprehension of disordered immune function and genetic predisposition. Early intervention and better prognosis necessitate an early diagnosis and surveillance, as well as established guidelines for management.

## 4. Conclusion

We reported a rare case of MN with AIH–PBC overlap syndrome, suggesting a common pathogenesis. Our case aims to offer valuable insights and resources for individuals facing comparable clinical scenarios. Further research ought to focus on the pathogenesis, treatment, and prognostic factors of overlap syndrome to improve efficacy and outcomes.

Supplemental Digital Contents are available for this article (https://links.lww.com/MD/P500; https://links.lww.com/MD/P501).

### Acknowledgments

We are grateful to the medical team and all involved healthcare professionals for their collaboration and support in preparing this case report.

## Author contributions

**Supervision:** Miaoxia He, Xiaobin Mei.

**Writing – original draft:** Xiwen Lei.

**Writing – review & editing:** Rui Dong, Jing Sun.

## Supplementary Material


