# Supplementary figures and images for: A case of membranous nephropathy complicated by autoimmune hepatitis and primary biliary cholangitis
Source: Medicine (Baltimore). 2025 Aug 1;104(31):e42770. doi: 10.1097/MD.0000000000042770 (PMC12323911; doi:10.1097/MD.0000000000042770)

Figure 1

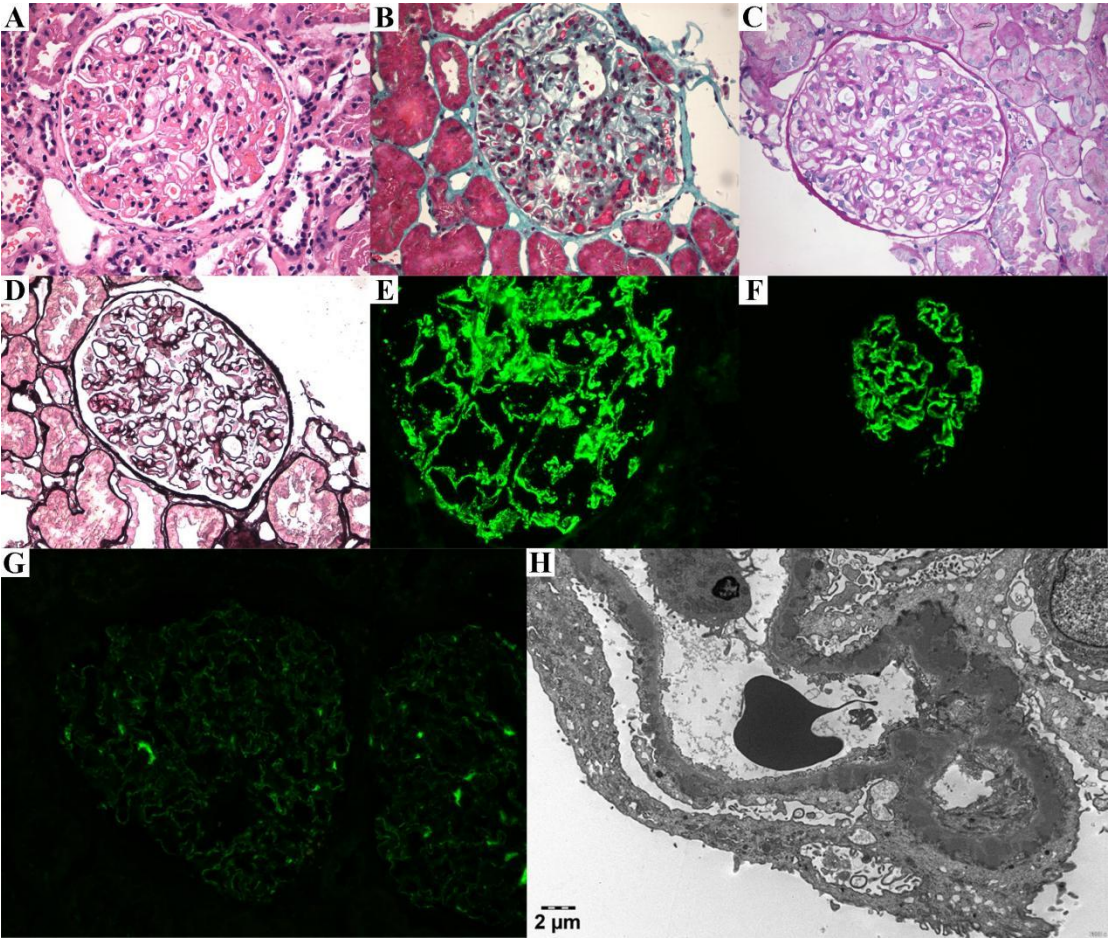

Figure 2

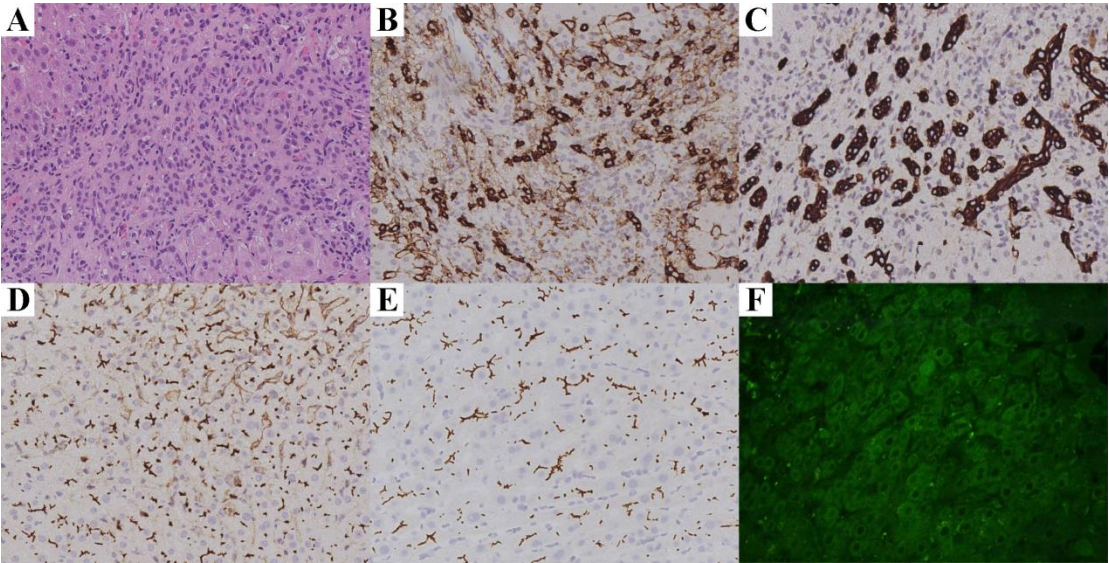

Supplement: Supplementary file 1 [file medi-104-e42770-s001.pdf]
