# Supplementary material for: A case of membranous nephropathy complicated by autoimmune hepatitis and primary biliary cholangitis
Source: Medicine (Baltimore). 2025 Aug 1;104(31):e42770. doi: 10.1097/MD.0000000000042770 (PMC12323911; doi:10.1097/MD.0000000000042770)
Supplement: Supplementary file 2 [file medi-104-e42770-s002.pdf]

Table. 1

Time Point	Investigations	Diagnosis	Interventions	Therapeutic Response
Pre-admission	PRO, 4+. OB, 4+.	Proteinuria and hepatic insufficiency.	Supportive treatment (not documented).	Symptoms resolved.
June 20, 2023 (Day 1)	ALB, 24 g/L. A/G, 0.88. TC, 8.3 mmol/L. LDL-C, 6.2 mmol/L. ALT, 161 U/L. γ-GT, 218 U/L. UP, 13245 mg/L. UPCR, 9195.5 mg/g. UACR, 7097 mg/g.	Proteinuria, hepatic impairment and hypertension.	Allisartan (240 mg/day), lercanidipine (10 mg/day), ezetimibe (10 mg/day), α-keto acid analogues (0.14 g/kg/day).	No prior issues with hypertension control.
July 3–13, 2023 (Days 14–24; first admission)	ALB, 23 g/L. A/G, 0.77. TC, 8.55 mmol/L. LDL-C, 5.62 mmol/L. ALT, 108 U/L. AST, 66 U/L. γ-GT, 194 U/L. IL-8, 22.76 pg/mL. anti-AMA-M2, positive then negative. anti-PLA2R antibody, 68.94 RU/mL. UP, 3393.0 mg/L. UPCR, 2506.1 mg/g. UACR, 1569 mg/g. UCa/Cr, 0.24 mg/g. Tf, 61.10 mg/L. κ-LC, 8.72 mg/L. λ-LC, 6.55 mg/L. UIgG, 37.9 mg/L. Urinary protein excretion/24h, 8191.8 mg. Urinary albumin excretion/24h, 6615.5 mg.	Nephrotic syndrome secondary to clinically suspected membranous nephropathy (PLA2R-positive), hepatic impairment and hypertension.	Renal biopsy, ezetimibe (10 mg/day), allisartan (240 mg/day), α-keto acid analogues (0.14 g/kg/day), ursodeoxycholic acid (750 mg/day), diammonium glycyrrhizinate (450 mg/day), first intravenous dose of rituximab (1 g).	Partial immunological remission of membranous nephropathy.
July 26–28, 2023 (Days 37–39; second admission)	ALB, 20 g/L. A/G, 0.74. TC, 6.97 mmol/L. LDL-C, 4.75 mmol/L. γ-GT, 94 U/L. UP, 12983.0 mg/L. UPCR, 8485.1 mg/g. UACR, 4597 mg/g. UCa/Cr, 0.24 mg/g. Urinary protein	PLA2R-associated membranous nephropathy and hypertension.	allisartan (240 mg/day), ezetimibe (10 mg/day), rivaroxaban (10 mg/day), α-keto acid analogues (0.14 g/kg/day), second intravenous	

	excretion/24h, 9357.0 mg. Pathological diagnosis: membranous nephropathy stage Ⅱ.		dose of rituximab (1 g).	
January 19, 2024 (Day 214)	ALB, 20 g/L. ALT, 228 U/L. AST, 112 U/L. ALP, 133 U/L. UA, 534 μmol/L. TC, 6.71 mmol/L. UACR, 1964.6 mg/g. Urinary protein excretion/24h, 6898 mg. anti-PLA2R antibody, 7.94 RU/mL. Abdomen ultrasound: chronic liver damage.	PLA2R-associated membranous nephropathy, hepatic impairment and hypertension.	Polyene phosphatidylcholine and glutathione for hepatoprotection.	Limited hepatoprotective efficacy.
February 20, 2024 (Day 246)	ANA (nuclear homogeneous type), 1:30. anti-dsDNA, 10 IU/mL. ANuA, 3.4 U/mL. ACA IgG, 6.8 PL-U/mL. anti-β2GP1, 15.5 RU/mL. ALB, 22 g/L. ALT, 282 U/L. AST, 230 U/L. ALP, 104 U/L. UA, 446 μmol/L. TC, 10.08 mmol/L.	PLA2R-associated membranous nephropathy, hepatic impairment and hypertension.	Liver biopsy, hepatoprotective and diuretic therapy.	Transaminase levels partially normalized.
March 20, 2024 (Day 276)	Pathological diagnosis: Primary biliary cholangitis－autoimmune hepatitis overlap syndrome.	Autoimmune hepatitis, primary biliary cholangitis, PLA2R-associated membranous nephropathy and hypertension.	Oral methylprednisolone (32mg once daily)	
April 16－18, 2024 (Days 302－304; third admission)	ALB, 25 g/L. A/G, 1.09. ALT, 45 U/L. AST, 18 U/L. ALP, 65 U/L. γ-GT, 123 U/L. TC, 5.99 mmol/L. IL-8, 22.99 pg/mL. anti-PLA2R antibody, <2.0 RU/mL. UP, 1978 mg/L. UPCR, 2582.9 mg/g. UACR, 1949	PLA2R-associated membranous nephropathy, autoimmune hepatitis, primary biliary cholangitis and hypertension.	Allisartan (240 mg/day), ursodeoxycholic acid (750 mg/day), pantoprazole (40 mg/day), α-keto acid analogues (0.14 g/kg/day), rivaroxaban	Complete immunological remission of membranous nephropathy and partial remission of proteinuria.

	mg/g. κ -LC, 11.6 mg/L. λ -LC, 7.72 mg/L. UIgG, 32.1 mg/L. Urinary protein excretion/24h, 4450.5 mg.		(10mg/day), oral methylprednisolone (20mg once daily), third intravenous dose of rituximab (1 g).	
